# Current Approaches and Molecular Mechanisms for Directly Reprogramming Fibroblasts Into Neurons and Dopamine Neurons

**DOI:** 10.3389/fnagi.2021.738529

**Published:** 2021-09-29

**Authors:** Fabin Han, Yanming Liu, Jin Huang, Xiaoping Zhang, Chuanfei Wei

**Affiliations:** ^1^Innovation Institute for Traditional Chinese Medicine, Shandong University of Traditional Chinese Medicine, Jinan, China; ^2^Shenzhen Research Institute of Shandong University, Jinan, China; ^3^The Institute for Tissue Engineering and Regenerative Medicine, Liaocheng University/Liaocheng People’s Hospital, Liaocheng, China; ^4^Laboratory of Basic Medical Research, Medical Centre of PLA Strategic Support Force, Beijing, China; ^5^College of Pharmacy, Shandong University of Traditional Chinese Medicine, Jinan, China

**Keywords:** Parkinson’s disease, fibroblast, direct reprogramming, transcription factor, miRNA, induced dopamine neuron

## Abstract

Parkinson’s disease is mainly caused by specific degeneration of dopaminergic neurons (DA neurons) in the substantia nigra of the middle brain. Over the past two decades, transplantation of neural stem cells (NSCs) from fetal brain-derived neural stem cells (fNSCs), human embryonic stem cells (hESCs), and induced pluripotent stem cells (iPSCs) has been shown to improve the symptoms of motor dysfunction in Parkinson’s disease (PD) animal models and PD patients significantly. However, there are ethical concerns with fNSCs and hESCs and there is an issue of rejection by the immune system, and the iPSCs may involve tumorigenicity caused by the integration of the transgenes. Recent studies have shown that somatic fibroblasts can be directly reprogrammed to NSCs, neurons, and specific dopamine neurons. Directly induced neurons (iN) or induced DA neurons (iDANs) from somatic fibroblasts have several advantages over iPSC cells. The neurons produced by direct transdifferentiation do not pass through a pluripotent state. Therefore, direct reprogramming can generate patient-specific cells, and it can overcome the safety problems of rejection by the immune system and teratoma formation related to hESCs and iPSCs. However, there are some critical issues such as the low efficiency of direct reprogramming, biological functions, and risks from the directly converted neurons, which hinder their clinical applications. Here, the recent progress in methods, mechanisms, and future challenges of directly reprogramming somatic fibroblasts into neurons or dopamine neurons were summarized to speed up the clinical translation of these directly converted neural cells to treat PD and other neurodegenerative diseases.

## Introduction

Parkinson’s disease is mainly caused by the degenerative loss of dopamine neurons (DA neurons) in the substantia nigra of the midbrain. Its characteristic biomarker is the formation of the Lewy body, which is composed of aggregated proteins in degenerating dopamine neurons and glial cells. Dopamine replacement drugs only delay the progression of the disease without curing the patients. Therefore, transplanting cells to replace damaged DA neurons has great potential in treating symptoms of Parkinson’s disease (PD) (Hu et al., 2015; Waldthaler and Timmermann, 2019; Wu et al., 2020).

Early studies transplanted human fetal brain-derived midbrain tissue or fetal neural stem cells (fNSCs) to PD patients and achieved some therapeutic effects (Lindvall et al., 1990; Freed et al., 2001; Li et al., 2008). Transplanted fNSCs have continuously improved the symptoms of PD patients, and they have survived in the brains of some patients for 16 years or even more than 24 years (Li et al., 2016; Xu et al., 2020). Other stem cells, such as embryonic stem cells (ESC), mesenchymal stem cells (MSC), and dopaminergic precursor cells, have been used to treat PD in preclinical studies (Han et al., 2015a; Cyranoski, 2017; Studer, 2017; Takahashi, 2017). The generation of autologous induced pluripotent stem cells (iPSCs) overcomes the rejection of other stem cells by the immune system, and it has broad clinical application in cell therapy and regenerative medicine (Han et al., 2015a, 2019). However, iPSC-derived neural stem cells and dopamine neurons may contain some residual undifferentiated cells and foreign gene integration, so iPSCs may pose a risk of tumorigenicity (Miura et al., 2009; Zhang et al., 2018; Barker and TRANSEURO Consortium, 2019; Ma, 2019). To overcome these obstacles, recent studies have explored directly reprogramming neurons or dopamine neurons from fibroblasts for transplantation therapy in PD (Fong et al., 2010).

Direct reprogramming, also known as transdifferentiation (Hou and Lu, 2016), uses exogenous genes to transform a mature somatic cell into another type of cell directly without going through the pluripotent stage. This approach is based on the reprogramming technology of iPS cells. In 2006, (Takahashi and Yamanaka, 2006) first reprogrammed mouse skin fibroblasts to iPS cells by retrovirus-mediated expression of four transcription factors, namely, Oct3/4, Sox2, Klf4, and c-Myc. In 2007, Yamanaka lab and Thomson lab reported the successful generation of human iPS cells by retrovirus-mediated transfection of OCT3/4, SOX2, KLF4, and c-MYC or lentiviral-mediated expression of four factors (OCT4, SOX2, NANOG, and LIN28) in human skin fibroblasts (Yu et al., 2007). Later, iPS cell-derived neural stem cells were reported to improve functions in animal models with PD, Alzheimer’s disease, and amyotrophic lateral sclerosis (ALS) (Han et al., 2015b, 2019; Bahmad et al., 2017). Furthermore, iPS cell-derived dopaminergic precursor cells or DA neurons were shown to improve the functional deficits of the primate model with PD (Hallett et al., 2015; Kikuchi et al., 2017).

Direct reprogramming of somatic cells mainly changes the expression of lineage-specific genes of the original cells through different epigenetic modifications without changing the genomic sequences of the genes. Mouse embryonic fibroblasts (MEFs) and human fibroblasts are the most commonly used starting cells for direct reprogramming to generate neural stem cells or neurons (Caiazzo et al., 2011).

**FIGURE 1:**
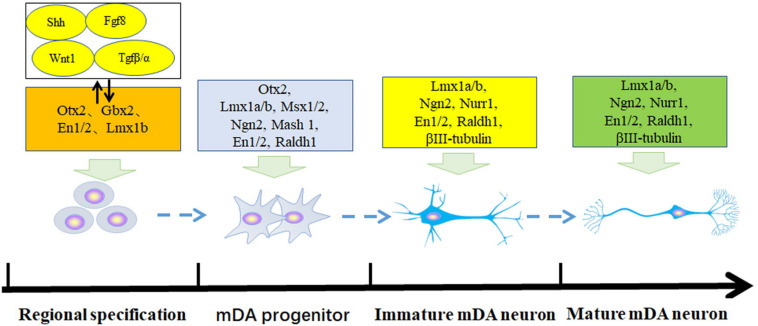
Transcriptional regulation of embryonic stem cells into mesencephalic dopamine progenitor and mature dopamine neurons. The development of dopamine neurons has four stages: regional neural specification, middle brain dopaminergic precursor cells, immature dopamine neurons, and immature dopamine neurons. These are controlled by different transcription factors.

The adult brain is composed of various neurons that are differentiated from neural precursor cells or neural stem cells. The maturation and differentiation of specific neurons, such as dopaminergic neurons, are involved in a series of complex and orderly gene expression and regulation processes. The developmental process of dopaminergic neurons *in vivo* has four main stages: (1) embryonic stem cells are induced into neural stem cells under the synergistic effect of transcription factor genes such as Ascl1, Lmx1a/b, Ngn2, En1/2, Nurr1, and Pitx3, (2) neural stem cells differentiate to the neurons or dopaminergic neuron lineage leading to the generation of early dopaminergic precursor cells, (3) early immature dopaminergic neurons are produced, and (4) at the same time the transcription factors act on the expression of the dopamine neuron-specific tyrosine hydroxylase (TH) or dopamine active transporter (DAT) genes to promote the maturation of dopamine neurons as shown in Figure 1 (Ang, 2006; Van Heesbeen et al., 2013; Veenvliet and Smidt, 2014; Arenas et al., 2015; Han et al., 2015b; Chen et al., 2020).

Based on the lineage-determining transcription regulatory networks in neural differentiation, different approaches have been developed to convert the fibroblast cells to neurons or DA neurons directly. They do this through epigenetic modification of genomic DNA, including methylation, histone modification, and nucleosome localization, and by repressing and activating the key regulating genes in determining the neural lineages (Graf, 2011; Xie et al., 2013; Mitchell et al., 2014; De Gregorio et al., 2018; Della Valle et al., 2020).

## Current Methods to Reprogram Somatic Fibroblasts to Neurons and Dopamine Neurons Directly

**FIGURE 2:**
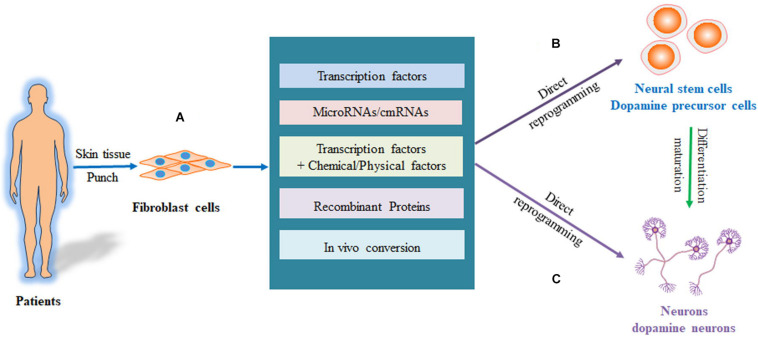
Process of directly reprogramming fibroblasts into neurons and DA neurons. **(A)** The fibroblasts are isolated from patients and reprogrammed to iPS cells, which are then differentiated into neurons or DA neurons. **(B)** The fibroblasts are directly converted to neural stem cells or dopaminergic precursor cells, which are then induced into neurons or DA neurons. **(C)** The fibroblasts can be directly reprogrammed into neurons or DA neurons. DA, dopaminergic neurons; iPS cells, pluripotent stem cells.

The cell fate is mainly controlled by endogenous lineage pathways and epigenetic modifications. Thus, the exogenous expression of the transcriptional factors to regulate the fate of the cell induces the direct conversion of somatic fibroblasts to neurons or dopamine neurons. Various approaches have been developed to convert fibroblasts to neurons or dopamine neurons. These include overexpression of transcription factors, RNA-based regulation of transcription factors, combining transcription factors with small-molecule compounds or other physical factors, and protein-based and *in vivo* approaches to induce epigenetic modification. These are summarized in Figure 2.

### Transcription Factor (TF)-Induced Direct Reprogramming of Fibroblasts to Neurons/Dopamine Neurons

Yamanaka et al. used four transcription factors, namely, OCT4, SOX2, c-MYC, and KLF4, to reprogram fibroblasts into iPS cells, showing that one cell type can be transdifferentiated to another type (Takahashi and Yamanaka, 2006). Like iPSC technology, most direct reprogramming also forced expression of the defined transcription factors to induce cell-fate conversion. Vierbuchen et al. (2010) first overexpressed transcription factors of Ascl1, Brn2, and Mytl1 to induce MEFs into mature neurons directly. This conversion process jumped over the state of pluripotent stem cells to convert mouse fibroblasts into functional neurons directly (Vierbuchen et al., 2010).

Shortly after that, Pfisterer et al. (2011) showed that the combined expression of the Ascl1, Brn2, and Myt1l with two additional Lmx1a and Foxa2 genes induced fibroblasts to specific iDANs with functional electrophysiological properties. They found that the combination of three factors, Ascl1, Brn2, and Myt1l, could convert fibroblasts to more than 95% of the neurons expressing the neuron marker MAP2 and more than 90% of the converted neurons expressed synaptophysin. This indicated that synapses were forming among the induced neurons (iN) (Pfisterer et al., 2011). In addition, the average resting membrane potential of the induced neurons was approximately −59 mV (range: −30 to −78 mV) at 30–32 days after transduction of the genes, indicating these converted cells exhibited the similar electrophysiological properties of functional neurons.

To induce specific DA neurons, they initially identified 10 genes involved in the specification of dopamine neurons in the midbrain (En1, Foxa2, Gli1, Lmx1a, Lmx1b, Msx1, Nurr1, Otx2, Pax2, and Pax5). These genes were ever thought to be required for converting the fibroblasts into induced neurons (iNs). Of these converted neurons, some also expressed the dopaminergic marker, TH, indicating that some iN cells were specified to dopamine neurons. They further determined that the minimal required genes are Lmx1a and Foxa2, which are co-expressed with the factors Ascl1, Brn2, and Myt1l, to induce the fibroblasts to dopaminergic neurons. These iDA neurons were found to have morphological and electrophysiological characteristics similar to dopaminergic neurons from fetal brain tissues. These iDAs were shown to co-express TH, Aromatic l-amino acid decarboxylase (AADC), the second enzyme in dopamine synthesis, and Nurr1 expressed by midbrain dopaminergic neurons. In the meantime, Caiazzo et al. (2011) directly reprogrammed human and mouse embryonic fibroblasts to functional dopamine neurons with a conversion efficiency of 18 ± 3%. They did this by combining three transcription factors: Ascl1, Nurr1, and Lmx1a. High-performance liquid chromatography showed that the iDAs released dopamine transmitters into the culture medium even though the iDAs still had some gene expression profiles different from midbrain-derived dopamine neurons (Caiazzo et al., 2011). Jongpil Kim et al. included sonic hedgehog (SHH) and fibroblast growth factor 8 (FGF8) proteins in the induction medium to promote the differentiation and maturation of iDAs. As a result, a combination of Ascl1, Nurr1, Pitx3, Lmx1a, Foxa2, and EN1 with SHH and FGF8 significantly enhanced the efficiency of EGFP^+^ iDAs from 8% on day 12 to 9.1% by day 18. After transplantation, the iDAs survived in the brain of the PD mouse model and improved the symptoms of dyskinesia in the PD mouse model (Kim et al., 2011; Liu et al., 2012).

Oh et al. (2014) efficiently reprogrammed mouse fibroblasts into a variety of neuronal cells including dopaminergic neurons. They found that after overexpressing the transcription factors Ascl1 and Nurr1 in MEFs, dopaminergic neurons were obtained by culturing the induced cells with growth factors such as SHH and FGF8 (Oh et al., 2014). They reduced the number of transcription factors and promoted the conversion of dopamine neurons from MEFs by modifying the culture conditions. This showed that the required transcription factors can be reduced and the efficiency of converting iDANs can be increased by changing the conditions of the induction culture. The Jialin Zheng team used another combination of transcription factors, namely, Brn2, Sox2, and Foxa2, to reprogram mouse embryonic fibroblasts and human fibroblasts directly into dopaminergic precursor cells. They showed that the dopaminergic precursor cells did not differentiate into astrocytes, and they did not form tumors. These studies suggest that induced dopamine precursor cells derived from direct reprogramming can be used for transplantation treatment of PD (Tian et al., 2015; He et al., 2019). It was also reported that induced noradrenergic (iNA) neurons can be induced from mouse astrocytes and human fibroblast cells by direct reprogramming of seven transcription factors (TFs) (Ascl1, Phox2b, AP-2α, Gata3, Hand2, Nurr1, and Phox2a). These iNA neurons function to release, and re-uptake of noradrenaline and can survive and form synaptic connections after transplantation to mouse model with neurological disorder (Li et al., 2019). The transcription factors used to induce fibroblasts to neurons or dopamine neurons by direct reprogramming are summarized in Table 1.

**TABLE 1:** The transdifferentiation of fibroblasts to neurons and dopamine neurons by direct reprogramming.

Source cells	Induced cells	Transcription factors/miRNA/chemicals	Efficiency	Transplantation	Investigators	PMID
Mouse embryonic and postnatal fibroblasts	Functional neurons	Ascl1, Brn2, and Myt1l	19.55% neurons	No	Vierbuchen et al., 2010	20107439
Human fibroblasts	Dopaminergic neurons	Ascl1, Brn2, Myt11, Lmx1a, FoxA2	16% neurons; 4.3% DA neurons	No	Pfisterer et al., 2011	21646515
Mouse and human fibroblasts	Functional dopaminergic neurons	Mash1 (Ascl1), Nurr1 (Nr4a2), and Lmx1a	10% neurons; 5% DA neurons	No	Caiazzo et al., 2011	21725324
Human fetal lung fibroblasts	DA neurons	Mash1, Ngn2, Sox2, Nurr1, and Pitx3	Less than 4% DA neurons	Yes	Liu et al., 2012	22105488
Mouse fibroblasts	Induced neurons and DA neurons	Ascl1 and Nurr1 and neurotrophic factors including SHH and FGF8b	51% Tuj1^+^ neurons, 33% DA neurons	No	Oh et al., 2014	24991651
Human embryonic fibroblasts	Induced neurons	Ascl1,Brn2,Myt1l, small molecules	MAp2 ^+^ neurons 46%	Yes	Pereira et al., 2014	25208484
Mouse fibroblasts	Dopaminergic precursors	Brn2, Sox2 and Foxa2, SHH, and FGF8	TH^+^ 90% of the Tuj1^+^ neurons	Yes	Tian et al., 2015	26224135
Human fetal lung fibroblast	Dopaminergic neurons	Ascl1, Nurr1, Lmx1a and miR124, p53 shRNA	Tuj1^+^ cells 31.1 ± 1.9%; TH^+^ cells (15.4 ± 1.1%)	No	Jiang et al., 2015	26639555
Human fibroblasts	Neurons/dopaminergic neurons	Brn2, Sox2, and Foxa2	96.67 and 86.75% expressing TH^+^/NeuN^+^ and TH^+^/MAP2^+^	No	He et al., 2019	30664902
Mouse and human Astrocytes and Foreskin fibroblasts	Functional noradrenergic neurons	Ascl1, Phox2b, Ap-2a, Gata3, Hand2, Nurr1, andPhox2a	6.6 ± 1.0%TH^+^ neuron	Yes	Li et al., 2019	31315047
Human postnatal and adult fibroblasts	Striatal neurons, GABAergic neurons	miR-9/9*-124 with CTIP2 (BCL11B) DLX1, DLX2, MYT1L	90% MAP2^+^, and 84% NeuN^+^; 72.3% GABAergic neurons of MAP2^+^ cells	Yes	Victor et al., 2014	25374357
Human fetal lung fibroblast	Neurons/iDA neurons	miR124, p53 shRNA, Ascl1, Nurr1, and Lmx1a	31.1% Tuj1^+^ cells, 15.4% TH^+^ cells	No	Jiang et al., 2015	26639555
Human dermal fibroblasts	Neural precursor cells, GABAergic or glutamatergic neurons	cmRNA SOX2 and cmRNA PAX6	38% TUJ1^+^ neurons	No	Connor et al., 2018	30450440
Mouse embryonic fibroblasts and adult tail-tip fibroblasts	Neural stem cells, neurons	Valproic acid, Bix01294, RG108, PD0325901, CHIR99021, Vitamin C, and A83-01 treatment	31% neurons expressing MAP2^+^	Yes	Han et al., 2016	26788068
Mouse fibroblasts	Neuron-like cells	VPA, TD114-2 (CHIR99021), 616452, Tranylcypromine, Forskolin, AM580, EPZ004777	50% of cells expressing tau, Tuj1 and MAP2	Yes	Li et al., 2017	28648365
Human dermal fibroblasts	Neuron-like cells	Ngn2 and Ascl1; LDN-193189, A83-1, CHIR99021, Forskolin, SB-431542, Pyrintegrin, ZM336372, AZ960, and KC7F2	80% of cells expressing Tuj1 and NeuN	No	Herdy et al., 2019	31099332
Fibroblasts	Induced neuronal cells	Fusion protein of 30Kc19 and Ascl1	Not indicated	No	Ryu et al., 2020	32058047
Mouse embryo fibroblasts	Induced neuronal cells	PTB-shRNA	Not indicated	No		23313552
Mouse astrocytes *in vivo*	Induced dopamine neuron	NEUROD1, ASCL1 and LMX1A, and miR218	30.97% neurons and 16% induced dopamine neuron	Yes	Rivetti Di Val Cervo et al., 2017	28398344
Mouse muller glia and astrocytes in striatum mouse	Induced neuron conversion	CasRx gene with two gRNAs 5 and 6 to knockdown Ptbp1	32% TH^+^ cells	Yes	Zhou et al., 2020	32272060
Astrocytes in the middle brain	Neurons, DA neurons	PTB-shRNA	80% of cells expressing NeuN; 22% of cell expressed TH and GIRK2 (A9 DA neurons)	No	Qian H. et al., 2020	32581380

### RNA-Mediated Direct Reprogramming

RNA is another approach to mediate reprogramming fibroblasts directly to neurons. This approach can eliminate the risk of mutations from integrating and inserting plasmid-based foreign genes and thus has the good potential use for clinical application of the converted cells (Ambasudhan et al., 2011; Yoo et al., 2011; Liu and Verma, 2015). There are two methods for RNA-based direct reprogramming.

#### Direct Reprogramming Mediated by MicroRNA (miRNAs)

The ectopic expression of miRNAs (miR-9/9^∗^ and miR-124) was shown to promote the direct conversion of human fibroblasts into neurons. To generate more-specific neuronal types, miR-9/9^∗^ and miR-124 were co-expressed with the striatum-enriched transcription factors, BCL11B, DLX1, DLX2, and Myt1l to induce human postnatal and adult fibroblasts into neurons that were similar to striatal spiny neurons (MSNs). In addition, after being transplanted into the mouse striatum, these converted neurons displayed functional properties similar to native MSNs. This study suggests that directly reprogrammed neurons might also be used in transplantation therapy of PD (Victor et al., 2014).

To increase the efficiency of converting fibroblasts to iDANs by RNA-based reprogramming, Jiang et al. (2015) combined three transcription factors (Ascl1, Nurr1, and Lmx1a) with miR124 and p53-shRNA to knockdown p53 synergistically and induce human embryonic lung fibroblasts to iDANs. They found that inhibiting p53 can arrest the cell cycle in the G1 phase and significantly increase the conversion of human fibroblasts to dopamine neurons. The efficiency of converting Tuj1^+^ neurons was increased from 17.9 to 31.1% and the efficiency of converting TH^+^ iDANs was increased from 8.3 to 15.4% (Jiang et al., 2015). Pu et al. (2020) used miR124 and p53-shRNA in a combination of transcription factors to reprogram the patient’s fibroblasts with Parkin gene mutation to the induced neurons. They found that mutations in the Parkin gene reduced the efficiency of converting somatic fibroblasts to TH + dopamine neurons (8.3 ± 0.6%), but did not affect the conversion efficiency of fibroblasts to total Tuj1 + neurons (18.2 ± 1.2%) (Pu et al., 2020). De Gregorio et al. (2018) used miR-34b/c combined with the transcription factors Ascl1 and NURR1 to reprogram fibroblasts into dopamine neurons directly with an efficiency of 19.5%. They showed that miR-34b/c helped cells to leave the cell cycle by regulating the expression of WNT1, and this greatly improved reprogramming efficiency. The iDANs have also been shown to synthesize dopamine, and they are consistent with the electrophysiological properties of dopaminergic neurons of the human midbrain (De Gregorio et al., 2018).

#### Direct Reprogramming Mediated by Chemically Modified mRNA (cmRNA)

In 2018, for the first time, Bronwen Connor et al. (2018) developed a method to transiently express the neural transcription factors SOX2 and PAX6 through cmRNA to induce neural stem cells from adult human fibroblasts directly. Transfecting fibroblasts with cmRNA-expressing SOX2 and PAX6 resulted in a higher conversion of fibroblasts to NSCs, and it improved cell survival. Furthermore, the cmRNA-induced NSCs were shown to differentiate to GABAergic and glutamatergic neuronal phenotypes to form in conjunction with astrocytes. The same research team also used cmRNA to reprogram adult fibroblasts into intermediate neural precursor cells (iNPs) and to induce iNPs to dopamine neuron-like cells expressing TUJ1, TH, AADC, DAT, VMAT2, and GIRK2 *via* (Playne et al., 2018). Compared with plasmid transfection, cmRNA has the advantage of being extremely stable and non-immunogenic. This makes it clinically applicable for gene delivery to generate directly induced neurons to treat neurological diseases.

### Direct Reprogramming Mediated by the Combination of Transcription Factors and Small-Molecule Compounds

Small molecules were reported to promote the neural conversion of fibroblasts by inhibiting or activating the signal pathways that regulate the neuronal lineage fate. Ladewig et al. (2012) demonstrated that the combination of transcription factors Ascl1 and Ngn2 with the small-molecule compounds, GSK-3β, SB431542, CHIR99021, Noggin, and LDN193189 converted human fibroblasts into functional neuronal cells. By inhibiting the SMAD signaling pathway, small molecules significantly improved the converted efficiency of iNs to 80% compared with the approach mediated by transcription factors (Ladewig et al., 2012). Pereira et al. (2014) co-expressed transcription factors of Ascl1, Brn2, Myt1l, Lmx1a, Lmx1b, Foxa2, and Otx2 in human embryonic fibroblasts (HEF) with small-molecule compounds (CHIR99021, SB431542, Noggin, LDN193189) to convert HEF into specific dopamine neurons efficiently (Pereira et al., 2014). The combination of small molecules with transcription factors reduces the number of required transcription factor genes for direct reprogramming. On the other hand, it can also decrease the potential risk of gene mutation and tumorigenicity caused by integrating foreign genes into the chromosomes of somatic cells (Wang et al., 2016).

Some studies used only chemical compounds for the direct conversion of fibroblasts to neurons without transcription factors. In 2015, Deng et al. reported that using only small molecule compounds could directly reprogram MEFs into functional neurons (Li et al., 2015). In the same year, Wenxiang Hu et al. used seven small molecules of valproic acid (VPA), CHIR99021, Repsox, Forskolin, SP600125 (a JNK inhibitor), GO6983 (a PKC inhibitor), and Y-27632 (ROCK inhibitor) to convert human fibroblasts directly into neuronal cells expressing doublecortin (Dcx), neuron-specific class III ß-tubulin (Tuj1), and microtubule-associated protein 2 (Map2). These chemical-induced human neuronal cells resembled the human iPSC-derived neurons in terms of morphology, gene expression, and electrophysiological properties (Hu et al., 2015). Then, Wang et al. (2016) used small molecule compounds (SB431542, Noggin, RA) and specific growth factors (SHH, bFGF, EGF, GDNF, and FGF8b) to convert MEF cells into dopamine neurons efficiently. Yang et al. (2019) used small-molecule compound combinations to induce human skin fibroblasts into neural cells efficiently. After transplantation, the induced neural cells can form functional connections with host cells in immune-deficient mice (Yang et al., 2019). Of note, by now studies have reported the transdifferentiation of MEF cells into neurons or dopamine neurons using only the small molecules, but no adult fibroblasts have been converted to neurons only by small molecules. Some of the small molecules used indirectly can activate neuronal TFs, so this suggests that they do not do so at expression levels high enough to drive reprogramming. The small molecules, PD0325901, CHIR99021, and A83-01 were first used to start the neural induction of MEFs and mouse tail-tip fibroblasts (TTF) by regulating the fate of stem cells. Then, the small molecules valproic acid, Bix01294, and RG108 were added to the medium to improve the induction of NSC and decrease the apoptosis of cells induced by vitamin C during the induction. These direct induced cells expressed the NSC markers of sex-determining region Y box-2 SRY-Box Transcription Factor 2 (Sox2), glial fibrillary acidic protein (GFAP), and oligodendrocyte transcription factor (Olig2), and they could be differentiated further to 31% neurons expressing Map2 and glial cells including 20% of GFAP-positive cells (astrocytes), 36% of O4-positive cells (oligodendrocytes) (Han et al., 2016). Li et al. reported that a seven-compound cocktail [VPA, TD114-2 (CHIR99021), 616452, Tranylcypromine, Forskolin, AM580, EPZ004777] induced MEFs into extra-embryonic endoderm (XEN)-like cells. Then they used Dorsomorphin, LDN193189, SB431542, and Ch55 to promote XEN-like cells into neurons of which more than 50% expressed neuron-specific genes tau, Tuj1, and MAP2. By day 18, more than 80% of the tau-positive cells were differentiated neurons expressing vGLUT1. This study also showed that the XEN-like cells further differentiate to mature neurons after transplantation into the adult mouse brains (Li et al., 2017).

Physical factors also can be combined with transcription factors to induce direct reprogramming. Junsang Yoo et al. (2015) reprogrammed fibroblasts into dopamine neurons through nanoscale biophysical stimulation. The fibroblasts were cultured on flat microgrooved and nanogrooved substrates. After biophysical stimulation, the expression of DA markers was gradually detected in the induced fibroblasts. These neuron conversions by physical stimulation may be attributed to specific histone modifications and transcriptional changes associated with mesenchymal-to-epithelial transition (Yoo et al., 2015). Another study reported that the same transcription factors (Ascl1, Pitx3, Nurr1, and Lmx1a) with the use of electromagnetic gold nanoparticles can directly reprogram mouse tail fibroblasts and human fibroblasts into dopamine neurons with great efficiency (Yoo et al., 2017). Chang et al. (2018) used mesoporous silica nanoparticles as non-viral vectors to transduce three plasmids, pAscl1, pBrn2, and pMyt1l, into mouse fibroblasts to produce functional dopaminergic neuron-like cells with function similar to fetal brain-derived dopamine neurons.

### Protein-Mediated Reprogramming

In this approach, recombinant proteins are transferred to somatic cells directly to induce their transdifferentiation into neurons. Mirakhori et al. (2015) used *trans-*activator of transcription (TAT)-mediated recombinant proteins of the transcription factors SOX2 and LMX1a, which were transfected into human embryonic fibroblasts in combination with small-molecule compounds, to generate dopaminergic precursor-like cells (iDPCs). Then they directly differentiated them into dopamine neurons (Mirakhori et al., 2015). This was the first report that iDPCs could self-proliferate and were safe and that they might become an option for cell replacement therapy in PD. Ryu et al. (2020) used the fusion partner of 30Kc19 protein and transcription factor Ascl1 to induce fibroblasts directly into neurons. Although its conversion efficiency was lower than that of other methods, protein-based direct reprogramming avoids the potential risk of gene mutation caused by foreign genes, and it also shows good potential for clinical application (Ryu et al., 2020).

Recently, human fibroblasts were converted into DA neuron-like cells by a combination of proteins and small molecules. The fibroblasts were first induced in the neural induction medium with valproic acid (VPA), Repsox, kenpaullone, forskolin, L Y-27632, and purmorphamine for 6–8 days. Then the induction medium was replaced with neuronal maturation medium with forskolin, kenpaullone, L-ascorbic acid, SHH, FGF-8b, bFGF, BDNF, and GDNF for two more weeks. The fibroblasts were converted into the induced DA neuron-like cells that expressed DA markers, exhibited neuronal morphology, and showed electrophysiological properties like that of fetal DA neurons. Further analysis showed that about 87.9% of the cells were converted into TUJ1^+^/TH^+^ neurons, indicating high yields of DA neuron-like cells were possible after induction by small molecules and proteins (Qin et al., 2020).

### *In vivo* Direct Reprogramming

The exogenous transplantation of directly converted neurons from cultured fibroblasts faces the problem of rejection by the immune system. However, direct reprogramming of glial cells into neurons *in vivo* also has made significant progress for clinical translation. Retroviral vectors infect dividing cells like progenitor cells or reactive glial cells without infecting the neurons. When retroviral vectors expressing transcription factor NeuroD1 were injected directly into the adult mouse cortex, the astrocytes were reprogrammed into functional neurons, such as glutamatergic neurons *in vivo* (Guo et al., 2014). To produce the induced dopamine neurons *in vivo*, the astrocytes of the mouse brain were transfected with NEUROD1, Ascl1, and LMX1A. The miR218 (NeAL218) were converted into iDAs with an efficiency of 16%. Importantly, this combination of NeAL218 successfully converted the striatal astrocytes into iDAs in a mouse model of Parkinson’s disease, and it improved some motor deficits *in vivo*. This meant that this approach might be suitable for clinical therapies in PD (Rivetti Di Val Cervo et al., 2017).

As it was discussed above, different approaches have their advantages and shortages to directly reprogram one cell type to another. Here the pros and cons of direct reprogramming were summarized as Table 2.

**TABLE 2:** Comparison of the PROS and CONS of different reprogramming approaches.

Approaches	PROS	CONS
Transcription factors-mediated method	High transdifferentiation efficiency, More precisely control the directions of transdifferentiation	Lenti_viral transfection integrate the foreign genes to the cell genome to induce mutagenesis
Combination of transcription factors and small molecules	Improved the converted efficiency of iNs to 80%, Using the small molecules to regulate the target genes and reduce the number of transcription factors required for direct reprogramming	May have genomic integration of some transcription factor genes, May have the risk of mutagenesis in directly reprogrammed cells
Chemical-mediated method	No foreign gene integration into the genome to reduce the risk of mutagenesis, suitable for clinical application	Has lower reprogramming efficiency and induced neurons are not mature. No adult fibroblasts have been converted to neurons by small molecules only.
mRNA-mediated method	No foreign gene integration into the genome to reduce the risk of mutagenesis, being extremely stable and non-immunogenic, suitable for clinical application	The safety and efficacy of the induced neurons needs further approval
Protein-mediated method	No foreign gene integration into the genome to reduce the risk of mutagenesis, A good potential for clinical application	Has lower reprogramming efficiency

## The Molecular Mechanisms of Direct Reprogramming

To increase the efficiency of directly reprogramming fibroblasts into neurons or dopamine neurons, it is necessary to understand the molecular and cellular mechanisms of cell fate conversion. This includes the roles of transcription factors, the regulatory networks of the transcriptome, and epigenetic modification during different stages of cell transitions. These mechanisms are mainly involved in the direct conversion of fibroblasts to neural stem cells, neurons, and dopamine neurons as noted in the following subsections.

### Transcription Factors Induce Direct Reprogramming of Fibroblasts to Neurons and Dopamine Neurons

As noted above, most studies used three transcription factors, namely, Ascl1, Brn2, and Myt1l or Ascl1, Nurr1, and Lmx1a to induce converted neurons and dopamine neurons. Ascl1 seems to play a pivotal role in the direct reprogramming of fibroblasts into neurons. It might be that Ascl1 enters the nuclei of the cells and binds its neuronal target genes across the genome of the fibroblasts. Structural analysis showed that bHLH transcription factors like Ascl1 bind to the two neighboring major groves of target DNA sequences with its α-helical basic domains as a heterodimer to induce the epigenetic modification and neuronal conversion (Ma et al., 1994; Kim et al., 2009). One study investigated this mechanism by characterizing the Ascl1 binding sites in MEF cells. ChIP-seq analysis showed that after Ascl1 was transfected into MEF cells, the Ascl1 binding pattern of MEFs was identical to that for all three transcription factors (Ascl1, Brn2, and Myt1l). This suggests that Ascl1 binds the same critical sites in the promoter regions of the regulatory genes as the other two factors, Brn2 and Myt1l, to start the conversion of fibroblasts to neurons. This showed that exogenous Ascl1 binds to the specific DNA sequence sites in fibroblasts without association with Brn2 or Myt1l (Wapinski et al., 2013). To further characterize the binding sites of Ascl1, a genome-wide analysis found that different binding sites had an affinity comparable to that of Ascl1 in MEFs. Furthermore, the CAGCTG sequence was identified as the key motif in the binding site of Ascl1. Several target genes also were bound by Ascl1. Some of them are members of the notch and JAK/STAT signaling pathways such as Zfp238, *Hes6*, *Dll1*, and *Mfng*. Others are genes such as *NeuroD4* that are associated with neural development.

The binding sites of Brn2 were also analyzed in different cells by ChIP-seq. The results showed that there was little overlap in the binding sites of Brn2 between fibroblasts and neural precursor cells (NPCs). However, an E-box motif was significantly enriched in a large fraction of these binding sites. A comparison of Brn2 and Ascl1 targets in MEFs infected by Ascl1, Brn2, and Myt1l found a high overlap between the target sites of Ascl1 alone and of both Brn2 and Ascl1. This suggests that Ascl1 actively recruits Brn2 to its targets, and it was further confirmed by the co-localization of Brn2 and Ascl1. Ascl1 binding sites are similar in MEFs and NPCs. However, a small fraction of sites was bound by Ascl1 in NPCs that are not bound in MEFs. This suggests that Ascl1 binds to specific sites of MEFs to induce neuronal conversion. That study also explored more than 25 predicted target genes, and it identified the transcription factor Zfp238 as the critical target of Ascl1 to mediate the reprogramming of fibroblasts to iN, together with other unidentified critical downstream factors (Wapinski et al., 2013).

Interestingly, another study reported that ASCL1 alone is able to induce functional iN cells from mouse and human fibroblasts and embryonic stem cells, further indicating that ASCL1 is playing the key role for iN and that MYT1L and BRN2 is primarily to promote the neuronal maturation (Chanda et al., 2014).

To explore the epigenetic methylation in the transdifferentiation of fibroblasts, an epigenetic model of ChromHMM was used to define chromatin methylation and acylation in entire genomes of MEFs (Ernst and Kellis, 2012). That analysis identified a specific MEF chromatin state (ChromHMM state 5) that is enriched at Ascl1 binding sites. ChromHMM state 5 represents the combination of high enrichment values for H3K4me1 and H3K27ac and low-to-intermediate enrichment levels of H3K9me3 in MEFs. Among MEFs, NPCs, human fibroblasts, and keratinocytes, this trivalent chromatin state, composed of H3K4me1, H3K27ac, and H3K9me3, was confirmed to be the key histone modification in the genome. Of all chromatin hidden Markov model (ChromHMM) states, state 5 had the highest enrichment at Ascl1 targets. Sequential immunoprecipitation of mono-nucleosomes isolated from MEFs revealed the co-occurrence of the three chromatin states (H3K27ac, H3K4me1, and H3K9me3) on single nucleosomes. This validated the existence of this trivalent state in the MEFs.

Several studies showed that small-molecule compounds increase transdifferentiation by inhibiting several cell fate signaling pathways. They include TGF-b/SMAD signaling, GSK-3b signaling (Ladewig et al., 2012), adenylyl cyclase activation (Gascon et al., 2016), and the RE1-silencing transcription factor (REST) pathway (Masserdotti et al., 2015). Herdy et al. (2019) have converted the fibroblasts to neurons by expressing the two transcription factors of NGN2 and Ascl1 and including small molecules in the induction medium to get more than 90% induced neurons (iN) over the starting fibroblasts (Wendt et al., 2014).

Transcriptome analysis was performed at different time points of direct iN. It found that more than 500 pathways were significantly modified in the transdifferentiation process. The 10 most important pathways were characterized through 20 small molecules to activate or inhibit each of the pathways during conversion. As a result, four compounds were found to increase the conversion of positive iNs significantly. Each of these compounds promoted the conversion of young and old fibroblasts into iNs through different mechanisms. They were Pyrintegrin as integrin activator, AZ960 as Jak2 inhibitor, ZM336372 as Raf-1 activator, and KC7F2 as HIF1a inhibitor. Importantly, the combination of all four compounds (ZPAK) produced a higher yield of iN than any of the four compounds separately. This helped these compounds act through different pathways to induce conversion of fibroblasts to iN synergistically (Herdy et al., 2019).

The JAK/STAT signaling is involved in the reversible process of cell proliferation and differentiation. Inhibiting JAK2 was shown to force fibroblasts from the cell cycle and promote mesenchymal-to-epithelial transition (MET) and neuronal reprogramming of the fibroblasts. Fibroblasts exiting the cell cycle have been reported to improve the MET process. Moreover, adding AZ960 to inhibit Jak2, significantly decreased STAT3. This induced a more rapid switch to promote the MET in converted neurons (Wendt et al., 2014).

### Characterizing the Regulatory Genes and Signaling Pathways in Direct Reprogramming by Transcriptome Analysis (Rna-Seq)

To identify the transcriptional networks during direct conversion of fibroblasts into neurons, Treutlein et al. used single-cell RNA-seq to analyze multiple time points during the 22 day-process of reprogramming MEFs into induced neurons (iN) (Trapnell et al., 2014). They explored how individual fibroblasts responded to overexpression of Ascl1 during the initial phase of reprogramming and found that an expression threshold is required for Ascl1 to start direct reprogramming. Ascl1 is responsible for starting the expression of target genes to induce direct neural conversion. It was observed that over-expression of Ascl1 in MEFs upregulates neuronal target genes of Zfp238, Hes6, Atoh8 and downregulates cell cycle genes such as Birc5, Ube2c, and Hmga2. Most fibroblasts are found to be partially reprogrammed at the early phase, and later events are responsible for the complete reprogramming. Further analysis of the single MEF cells showed that in the first 5 days forced expression of Ascl1 is associated with increased expression of neuronal genes in reprogrammed MEF cells. Detailed time-lapse track of GFP-labeled single cells showed that some MEF cells with low or no Ascl1 expression in the converting process might never have been started for direct reprogramming (Treutlein et al., 2016).

During the 22-day process of converting MEFs into iN cells, a continuous intermediate process was observed at each time point through Monocle analysis of single converting cells (Trapnell et al., 2014). Principal component analysis (PCA) of all single cells in the early, intermediate, and late stages of neuronal transdifferentiation identified two genetic regulatory events during reprogramming. First, there is an initiation stage where MEFs exit the cell cycle upon Ascl1 induction, and regulating genes such as *Birc5*, *Ube2c*, *Hmga2* involved in mitosis are turned down or off. Concomitantly, the expression of some genes associated with cytoskeletal reorganization (*Sept3/4*, *Coro2b*, *Ank2*, *Mtap1a*, *Homer2*, and *Akap9*), synaptic transmission (*Snca*, *Stxbp1*, *Vamp2*, *Dmpk*, and *Ppp3ca*), and neural projection [*Cadm1*, *Dner*, *Klhl24*, *Tubb3*, and *Mapt (Tau)]* were all up regulated. This showed that Ascl1 induces the expression of genes involved in defining neuronal conversion during early reprogramming. This initiation phase is followed by a maturation stage in which extracellular matrix genes are turned off and the genes involved in synaptic maturation (*Syp*, *Rab3c*, *Gria2*, *Syt4*, *Nrxn3*, *Snap25*, and *Sv2a*) are turned on in converted MEF cells. These findings are consistent with the previous finding that Tuj1 + cells with immature neuron-like morphology could be found as early as the third day after Ascl1 is expressed, while functional synapses are not formed until 2 to 3 weeks after reprogramming begins (Xu et al., 2020).

### Retrotransposon-Mediated Conversion of Fibroblasts Into Dopamine Neurons

In mammalian cells, LINE-1 (L1) retrotransposons make up about 15 to 20% of the genomic DNA sequence. The mouse genome contains about a half-million L1 copies, but only 3,000 L1 copies can drive their expansion in the genome with a copy-paste mechanism. In the adult brain, L1 retrotransposons are preferentially expressed in some neural genes. Some L1 copies inserted in these genes induce deletions and replications of large genomic DNA fragments, and this inactivates some neural genes and induces the neural conversion of somatic cells (Upton et al., 2015; Erwin et al., 2016). Recently, Della Valle et al. (2020) reported that L1 reactivation mediated neuronal conversion through a conserved *de novo* insertion site in the expressed gene loci of iDANs. Through RNA-seq and transposase-accessible chromatin sequencing (ATAC-seq), they identified that, near L1 insertion sites, more non-coding RNAs (ncRNAs) are produced. This shows there is a correlation between L1 reactivation and cell lineage conversion (Caiazzo et al., 2011; Della Valle et al., 2020). In the direct conversion of MEF cells to iDANs through overexpression of three specific transcription factors, namely, Nurr1, Ascl1, and Lmx1a, L1 reactivation was confirmed by the production of full-length LINE-1 RNA in iDANs (Caiazzo et al., 2011). At the same time, the L1 copy number variation (CNV) was significantly increased in TH + iDANs. However, other retrotransposable elements, such as active and autonomous intracisternal A-type particle (IAP) elements and non-autonomous SINE B1 and B2 elements, were not induced in iDANs. That study provided evidence for the mechanism of L1 reactivation and CNV increase when mouse embryonic fibroblasts are converted into dopaminergic neurons.

### miRna-Mediated Mechanisms to Convert Fibroblasts to Neurons or DA Neurons

miRNAs are important for promoting the conversion of fibroblasts by regulating Wnt signaling and other pathways. Thus, regulating Wnt signaling has promoted the conversion of fibroblasts to mDA neurons *in vitro* (Nouri et al., 2015). Studies have shown that two miRNAs, miR-9/9^∗^ and miR-124, can convert fibroblasts into neurons. A further study reported that miR-9^∗^ and miR-124 induced the transdifferentiation of mouse fibroblasts to neurons by repressing the BAF53a subunit of the neural-progenitor (np) BAF chromatin-remodeling complex. This means that miR-9/9^∗^ and miR-124 (miR-9/9^∗^-124) are involved in regulating neuronal differentiation and miR-9/9^∗^-124 combined with NEUROD2 converted the human fibroblasts into neurons. It also showed that adding transcription factors, Ascl1 and Myt1l, increased the conversion rate and the maturation of the converted neurons, but expression of these transcription factors alone without miR-9/9^∗^-124 was relatively ineffective (Yoo et al., 2011). In addition, without miR-9/9^∗^-124, the same transcription factors hardly induce neuronal conversion. This means that miRNA might initiate neuronal states, and that transcription factors promote the maturation of converted neurons.

Further study showed that co-expression of miR-9/9^∗^-124 with the transcription factors BCL11B, DLX1, DLX2, and Myt1l enriched in the developing striatum, can convert human postnatal and adult fibroblasts into specific neurons analogous to striatal spiny neurons (MSNs) (Victor et al., 2014). Transcriptome analysis of starting fibroblasts and induced neurons showed the loss of fibroblast gene expressions such as S100A4, VIM, and COL13A1, and the gain of a pan-neuronal identity (MAP2, NEFL, SNAP25, and SCN1A) 35 days after the expression of miR-9/9^∗^-124 in fibroblasts. A time-lapse analysis of the transcriptome of cells undergoing a conversion induced by miR-9/9^∗^-124 showed that molecular pathways were potentially targeted by REST through the transcript regulation of REST (Abernathy et al., 2017).

Systematic analyses of the transcriptome, genome-wide DNA-methylation, and chromatin modification revealed that miR-9/9^∗^-124 can induce extensive modification of the epigenome and activate a neuronal lineage and the reconfiguration of chromatin accessibilities simultaneously. The miR-9/9^∗^-124 were shown to open the genomic loci for multiple subtype-specific genes, including established motor neuron markers. Therefore, it was postulated that specific neuron-enriched transcription factors would cooperate with miR-9/9^∗^-124 to specify a neuron subtype conversion of fibroblasts. To explore how miR-9/9^∗^-124 are involved in controlling REST expression to activate neuronal genes during neuronal reprogramming, it was found that repressing REST-activated chromatin opened regions that contained neuronal genes that encompassed binding sites of REST in the regulatory regions. Moreover, BAF53b was found to be a transcriptional target of REST and EZH2 repression, leading to REST destabilization to induce neuronal conversion. This is a conserved mechanism during neuronal differentiation of neural stem cells and developing mouse brain. This result showed that the activity of miRNAs represses EZH2-REST to promote the neuronal conversion of fibroblasts (Lee et al., 2018).

In a recent study, the miR-34b/c (miR-34b-5p/miR-34c-5p) were found to be involved in regulating the Wnt signaling pathway to increases *in vitro* differentiation of DA neurons significantly (De Gregorio et al., 2018). Thus, miR-34b/c was found to bind the *Wnt1 3’UTR* to reduce *Wnt1* directly in DA-differentiated mouse embryonic stem cells (mESCs). It was clearly shown that co-expression with Ascl1 and NURR1, miR-34b/c significantly increased the transdifferentiation efficiency of fibroblasts to iDANs with upregulated expression of DA markers such as Th, Nurr1, Pitx3, Dmrt5, Foxa2a, and Lmx1a. These iDANs were found to have specific electrophysiological properties as striatum DA neurons. In addition, microarray analysis of the iDANs revealed that five Wnt genes (Wnt1, Wnt5a, Wnt5b, Wnt7a, and Wnt9a) are downregulated in miR-34b/c-induced DA neurons from day 9 and day 14. This further suggests that miR-34b/c induced the transdifferentiation of fibroblasts to DA neurons through Wnt signaling. By transfecting plasmids expressing miR-34b/c and miR-148a-3p with a pmiR-luciferase reporter containing Wnt1 3-UTR, both miR-34b/c and miR-148a-3p could reduce luciferase activity significantly. This effect was repressed after mutation of the predicted binding site for miR-34b/c but not for miR-148a-3p. This suggests that only miR-34b/c effectively binds to its predicted site at 3’-UTR of Wnt1. To validate this result, the MEF cells derived from TH-GFP mice were infected with Ascl1, Nurr1, and miR-34b/c in the presence of CHIR99021, which can inhibit GSK3β to mimic Wnt activation. The result further confirmed that miR-34b/c induced transdifferentiation of fibroblasts to DA neurons by regulating Wnt signaling.

The mechanisms for *in vivo* direct reprogramming to convert glial cells to DA neurons are similar to that for direct conversion of the fibroblast cells to neurons or dopamine neurons *in vitro*. The commonly used cell source for brain repair is the astrocytes for direct reprogramming *in vivo* to generate DA neurons due to their wide distribution throughout the CNS. These mechanisms are mainly involved in the transcription factors, mRNAs, RNA interfere (RNAi) or miRNAs to regulate the specific genes such as for neuronal development and maturation (Rivetti Di Val Cervo et al., 2017; Wang et al., 2021) or to knockdown the specific genes such as the single RNA bing protein (Ptbp1) to directly reprogramming astrocytes to neurons *in vivo* (Zhou et al., 2020). A recent study has also systemically reviewed the progress and mechanisms of direct reprogramming *in vivo* (Qian C. et al., 2020).

## Conclusion

Direct reprogramming induces somatic cells to convert into functional neurons and dopamine neurons, which has great potential for drug screening and transplantation treatment of neurodegenerative diseases. The directly converted cells do not need to go through the state of pluripotent stem cells, avoiding the formation of tumors after being transplanted to the brain or other organs. Now iNs or iDANs can be easily achieved by forced expression of specific sets of transcription factors, miRNAs, or a combination with chemical compounds to activate or inhibit developmental lineages by regulating the Wnt, Notch, and JAK/STAT signal pathways.

Since the exogenous transcription factor genes may integrate into the host cell genome to induce genomic mutations in the directly reprogrammed cells (Pereira et al., 2019), future direct reprogramming can take advantage of the episomal plasmids to replace the viral vectors to express lineage-specific transcription factors without transgene integration. In addition, the use of RNA, miRNA, proteins, or small-molecule compounds is another safe way to generate clinically applicable neurons or dopamine neurons converted from fibroblasts.

When the induced neurons or dopamine neurons *in vitro* are transplanted to the brains, only a few percentage of these cells can survive for a long term to form synaptic connections with the host cells even though some studies reported that some levels of functional integration of the transplanted cells have been observed in host brains (Kim et al., 2011; Dell’anno et al., 2014). Thus *in vivo* transdifferentiation provides a significant advantage to induce the local glial cells of the brain to convert to the required neurons for treatment of neurodegenerative diseases such as PD or AD. A challenge for *in vivo* direct reprogramming is to know that the converted neurons are derived from local glial cells. One way to resolve this issue is to assess the functional improvement in neurological behavior abnormalities. Most recently, Qian H. et al. (2020) used a chemogenetic approach to improve the chemical-induced motor dysfunction of the mouse model of PD. This provided evidence for restoring neurological function by *in vivo* transdifferentiation. Another approach is to label the converted neuronal cells for *in vivo* tracking genetically (Rivetti Di Val Cervo et al., 2017; Qian H. et al., 2020).

Another problem facing direct reprogramming is the low efficiency of conversion and the poor survival of directly reprogrammed cells after transplantation. With increased understanding of the molecular mechanism and signaling pathways in the process of direct reprogramming, more efficient direct reprogramming methods will be developed to generate a large scale of induced neurons or dopamine neurons for clinical and research uses. In addition, efficient cell sorting technology will continue to be developed to purify the directly converted cells. In the future, skin fibroblasts will be directly reprogrammed to specific neurons, such as dopamine neurons, that are closer to their natural state, and they will provide sufficient cell sources for the treatment of PD and other neurodegenerative diseases by clinical transplantation.

## Author Contributions

FH contributed to the design, conceptualization, data collection, formal analysis, methodology, supervision, visualization, and writing the original draft. YL, XZ, JH, and CW contributed to the data collection, preparation of the figures and tables, and writing some of the manuscript. All authors read and approved the final manuscript.

## Conflict of Interest

The authors declare that the research was conducted in the absence of any commercial or financial relationships that could be construed as a potential conflict of interest.

## Publisher’s Note

All claims expressed in this article are solely those of the authors and do not necessarily represent those of their affiliated organizations, or those of the publisher, the editors and the reviewers. Any product that may be evaluated in this article, or claim that may be made by its manufacturer, is not guaranteed or endorsed by the publisher.

## Abbreviations

PD: Parkinson’s disease
iN: induced neuron
iDAN: induced DA neuron
NSC: neural stem cell
NPC: neural precursor cells
fNSC: fetal brain-derived neural stem cell
mESC: mouse embryonic stem cell
hESC: human embryonic stem cell
iPSC: induced pluripotent stem cell
MEF: mouse embryonic fibroblast
HEF: human embryonic fibroblast
TTF: tail-tip fibroblast
TF: transcription factor
CNV: copy number variation
ncRNA: non-coding RNA
L1: LINE-1
gRNAs: guide-RNAs
cmRNA: chemically modified mRNA.
